# Abnormal MRI Features in Children with ADHD: A Narrative Review of Large-Scale Studies

**DOI:** 10.3390/brainsci16010104

**Published:** 2026-01-18

**Authors:** Chunyang Wang, Shiyun Wang, Li Sun, Jing Sui

**Affiliations:** 1 State Key Laboratory of Cognitive Neuroscience and Learning, Beijing Normal University, Beijing 100875, China; wangchunyang@ldu.edu.cn (C.W.);; 2 School of Education, Ludong University, Yantai 264025, China; 3 Peking University Sixth Hospital, National Center of Mental Health, Beijing 100085, China

**Keywords:** attention-deficit/hyperactivity disorder, children, magnetic resonance imaging, brain structure, functional brain networks

## Abstract

Attention-deficit/hyperactivity disorder (ADHD) is a common neurodevelopmental disorder in childhood, characterized by persistent inattention, hyperactivity, and impulsivity. This narrative review aims to synthesize and critically evaluate recent large-scale magnetic resonance imaging (MRI) studies to clarify the neuroanatomical and functional brain alterations associated with ADHD in children. By addressing current gaps in understanding, this work seeks to identify reliable neurobiological markers that could improve diagnostic accuracy and guide personalized interventions. The literature reveals that large-scale structural MRI studies consistently report abnormal development in total cortical volume and surface area, prefrontal cortex volume, and basal ganglia volume in children with ADHD. Moreover, gray matter alterations show significant age-dependent effects, with the degree of impairment potentially serving as neurobiological markers. Diffusion magnetic resonance imaging studies reveal disrupted white matter microstructures in regions such as the left uncinate fasciculus, superior and inferior longitudinal fasciculi, corpus callosum, cingulum, and internal capsule. Importantly, these white matter abnormalities often persist into adulthood, highlighting their clinical relevance. Functional MRI findings indicate reduced global connectivity within core hubs of the default mode network in children with ADHD. Furthermore, deficits in inhibitory control identified via fMRI may represent one of the neurofunctional signatures that differentiates ADHD from typically developing controls. By consolidating evidence from large-scale multimodal MRI studies, this review provides a comprehensive understanding of the neurodevelopmental alterations in ADHD and underscores their potential utility for improving diagnosis and treatment.

## 1. Introduction

Attention-deficit/hyperactivity disorder (ADHD) is a neurodevelopmental disorder with onset in childhood, and symptoms may persist into adulthood [1,2]. Patients primarily exhibit developmentally inappropriate inattention and/or hyperactivity/impulsivity. Epidemiological data indicate that the prevalence of ADHD is 7.6% in children aged 3–12 years [3] and approximately 2.5% in adults [4]. During childhood, the prevalence of ADHD is significantly higher in boys than in girls; however, it is noteworthy that the prevalence in females is often underestimated due to lower rates of clinical consultation [5]. Childhood ADHD is not only highly prevalent but also frequently comorbid with other psychiatric disorders, such as learning disabilities, autism spectrum disorder [6], and oppositional defiant disorder [7].

In recent years, there has been substantial growth in large-scale magnetic resonance imaging (MRI) studies within the field of ADHD research. Despite this progress, comprehensive narrative reviews synthesizing findings from these large-scale MRI studies remain relatively limited. This gap is particularly noteworthy for two reasons. First, in neuroscience, findings from small-sample studies are often prone to low reproducibility [8,9,10,11], underscoring the critical need for integrative reviews that draw on large-scale data to provide more robust conclusions about ADHD-related neurobiology. Second, the clinical management of ADHD currently lacks objective neurobiological markers, which further highlights the importance of narrative reviews that integrate and critically evaluate large-scale MRI research across multiple modalities to elucidate the neuroimaging signatures of ADHD. In this review, large-scale studies on ADHD are operationally defined as those including a minimum of 60 children with ADHD, a minimum of 60 control participants, and a total sample size of at least 120, while employing rigorous methodological designs. This threshold was established to ensure adequate statistical power and reproducibility of findings. Accordingly, this review aims to examine the neurobiological substrates of childhood ADHD by synthesizing evidence across multiple imaging modalities, including structural MRI, diffusion MRI, and functional MRI.

## 2. Features of Gray Matter in Children with ADHD

Structural magnetic resonance imaging (sMRI), as an important MRI technique, provides a novel perspective for in-depth analysis of the neural mechanisms underlying ADHD. Research on the neural mechanisms of childhood ADHD based on sMRI technology has primarily focused on the whole-brain level (total cortical gray matter volume and total cortical surface area) as well as specific brain regions (cortical gray matter regions, subcortical nuclei, or subcortical regions). Current evidence suggests that the abnormal development of total cortical volume [12], the prefrontal cortex [13], the basal ganglia, and the hippocampus [14] plays a crucial role in the pathophysiological mechanisms of ADHD.

### 2.1. Total Cortical Volume and Total Cortical Surface Area

With the expansion of international ADHD consortium data [15]—characterized by its large scale, participant diversity, and strong sample representativeness—researchers have been able to more accurately describe structural brain differences in children with ADHD at the whole-brain scale through large-scale data sharing and analysis. Based on neuroimaging data analysis from 36 data centers worldwide, the study found [12] that intracranial volume in children with ADHD aged 4–14 years was significantly smaller than that of controls; however, no such significant differences were observed in individuals aged 15 years and older. Research based on the Adolescent Brain Cognitive Development (ABCD) study dataset [16] demonstrated that total intracranial volume in children patients was significantly smaller than that of healthy controls, and total cortical surface area was significantly reduced compared to healthy controls. Regarding different ADHD subtypes, a large-scale study based on the ADHD-200 dataset found that, compared with the typically developing group, the ADHD-Combined patients had significantly reduced cortical thickness of right hemisphere_caudalanteriorcingulate and right hemisphere _posteriorcingulate. ADHD-Inattentive patients had significantly reduced cortical thickness in the left hemisphere _posteriorcingulate and right hemisphere_lateraloccipital [17]. Structural MRI differences between children with ADHD and typically developing controls are shown in Table 1. Beyond changes in total cortical volume and total cortical surface area, large-scale studies suggest that abnormal prefrontal cortex development represents another important structural imaging feature of childhood ADHD.

**Table 1:** Differences in MRI features between ADHD and controls in large-scale studies.

MRI Features		Main Findings	Sample Size and Age		
			ADHD	Control	Age
sMRI	Total Cortical Surface Area	Significantly reduced total cortical surface area [16]	949	9787	9–10
		Significantly reduced total intracranial volume [12]	1081	1048	4–14
	Prefrontal Cortex	Significantly reduced prefrontal cortical thickness [18]	285	494	7–27
		Significantly reduced surface area in the dorsolateral prefrontal cortex [13]	244	277	8–12
	Hippocampus	Significantly reduced hippocampal volume [14]	764	802	4–14
	Basal Ganglia	Significantly reduced volumes in the amygdala, nucleus accumbens, caudate nucleus, and putamen [14]	767	820	4–14
dMRI	Corpus Callosum	Reduced FA in the splenium and body of the corpus callosum [19]	500	637	6–18
	Uncinate Fasciculus, Superior/Inferior Longitudinal Fasciculus	Reduced FA in the inferior longitudinal and left uncinate fasciculi [20]	951	4884	6–18
		Abnormal development of the superior longitudinal fasciculus [21]	99	85	9–14
		Altered FA in the bilateral inferior longitudinal fasciculus [22]	76	68	9–11
	Cingulum Angular Bundle	Lower FA in the right cingulum angular bundle was associated with higher hyperactivity–impulsivity symptom severity [23]	258	322	7–28
fMRI	rs-fMRI	Aberrant resting-state functional connectivity in core hub regions of the DMN [24]	227	227	7–18
		Increased resting-state functional connectivity between the striatum and temporal regions, as well as the supplementary motor area [25]	1696	6737	6–18
	task-fMRI	Reduced activation in the DMN, dorsal attention network, and limbic network during the Go/No-Go task [26]	224	232	≤18
		Aberrant activation levels in frontoparietal regions associated with response inhibition during the stop-signal task [27]	691	5110	9–11

### 2.2. Prefrontal Cortex

The prefrontal cortex (PFC) is a key brain region closely associated with higher-order cognitive functions in humans. In particular, the dorsolateral prefrontal cortex plays a crucial role in complex cognitive activities such as attention, working memory, rule learning, and decision-making [13]. Early research [28] indicated that gray matter volume in the prefrontal cortex of children with ADHD was smaller than that of typically developing children, and longitudinal study findings support this observation. A large-scale longitudinal study of children aged 5–14 years found [13] that prefrontal cortical thickness in children with ADHD was significantly reduced compared to age-matched typically developing controls, particularly in the dorsolateral prefrontal cortex and superior frontal cortex. Research findings based on large-scale global neuroimaging datasets indicate that in child populations (n = 2129), frontal cortical surface area in patients with ADHD was significantly smaller than that of typically developing controls; however, in adolescent and adult populations (n = 2051), no significant differences were found in either frontal cortical surface area or cortical thickness [12].

Overall, abnormal prefrontal gray matter development constitutes important evidence for the structural brain characteristics of childhood attention-deficit/hyperactivity disorder [18,29,30]. Further analysis reveals that prefrontal cortical developmental abnormalities in patients exhibit significant age effects [31]. During childhood, prefrontal cortical development in patients with ADHD is delayed compared to that of typically developing controls by 2–5 years [13], and by adolescence, this developmental delay gradually diminishes or disappears [12]. Behavioral research evidence also validates this conclusion [32,33,34].

### 2.3. Basal Ganglia, Hippocampus, and Cingulate Cortex

The basal ganglia are a group of subcortical nuclei located deep within the brain, primarily including the striatum (lentiform nucleus and caudate nucleus), nucleus accumbens, substantia nigra, subthalamic nucleus, and other nuclei. Research findings based on ENIGMA [14] demonstrate that in children with ADHD aged 4–14 years, volumes of the basal ganglia (nucleus accumbens, caudate nucleus, and putamen), amygdala, and hippocampus were significantly smaller than those of typically developing controls, with particularly pronounced volume reductions in the amygdala and nucleus accumbens. Recent research based on the ABCD dataset [16] found that the surface area of the caudal anterior cingulate cortex and posterior cingulate cortex in children with ADHD was significantly smaller than that of typically developing controls. Overall, the developmental level of subcortical brain regions (nuclei) in patients with ADHD also exhibits significant age-related characteristics, consistent with the developmental trajectory of cortical gray matter in patients [12].

## 3. Features of White Matter in Children with ADHD

Diffusion magnetic resonance imaging (dMRI) is a magnetic resonance imaging technique that can be used to probe the structural connectivity and microstructure of human brain tissue non-invasively in vivo. It is primarily used to investigate microstructural alterations in white matter tracts [35]. Commonly used quantitative indices include fractional anisotropy (FA) and mean diffusivity (MD). Changes in FA reflect white matter integrity under different pathological conditions; lower FA typically indicates damage to white matter microstructures or disruption of fiber tract coherence. MD reflects the extent of water diffusion within a voxel; higher MD values are generally suggestive of axonal or myelin injury in white matter [36]. Existing large-scale studies have demonstrated abnormal development of the left uncinate fasciculus, superior longitudinal fasciculus, and inferior longitudinal fasciculus in children with ADHD [20]. Meta-analyses have shown a consistent reduction in FA in the splenium and body of the corpus callosum [19]. Correlational analyses have shown that ADHD severity is associated with white matter microstructure in the subgenual cingulum [37].

### 3.1. Left Uncinate Fasciculus, Superior Longitudinal Fasciculus, and Inferior Longitudinal Fasciculus

The uncinate fasciculus (UF) connects the frontal lobe, temporal lobe, and parahippocampal gyrus, playing a key role in emotional–cognitive regulation and attentional control. The superior longitudinal fasciculus (SLF) is a major long-range white matter tract that links the frontal, parietal, temporal, and occipital lobes. The inferior longitudinal fasciculus (ILF) connects the anterior temporal lobe to the occipital lobe and forms indirect neural circuits with the frontal lobe via its connections with the uncinate fasciculus. A large-scale magnetic resonance imaging study (n = 951) reported significantly lower FA values in the left uncinate fasciculus and inferior longitudinal fasciculus in children with ADHD compared to typically developing controls [20], as summarized in Table 1. Further research has identified a significant negative correlation between FA in the bilateral inferior longitudinal fasciculi and ADHD symptom severity in pediatric populations [22]. Additionally, children with ADHD show markedly reduced FA in the superior longitudinal fasciculus relative to controls [38,39], with aberrant development of this tract consistently documented in ADHD patients [21].

### 3.2. Corpus Callosum

The corpus callosum is a critical structure connecting the left and right cerebral hemispheres and is responsible for interhemispheric information transfer. The corpus callosum participates in multiple important brain activities, including visual information integration, spatial perception, memory, higher-order cognitive functions, and emotional processing [19]. Its developmental abnormalities are associated with ADHD symptoms [40] and hold significant value in the clinical diagnosis of ADHD. Research has demonstrated that different subsegments of the corpus callosum exhibit microstructural developmental impairment in children with ADHD [41]. Specifically, FA values in the isthmus and posterior midbody of the corpus callosum are significantly lower in children with ADHD compared to typically developing controls [38], and FA values in the posterior corpus callosum connecting the temporal–parietal–occipital regions are significantly reduced [19].

### 3.3. Internal Capsule and Corona Radiata

The internal capsule is an important white matter structure located medial to the basal ganglia. The corona radiata is situated beneath the cerebral cortex, exhibits a radial distribution pattern, and connects the cerebral cortex with subcortical structures. Impaired development of white matter microstructures in the internal capsule or corona radiata is associated with multiple ADHD symptoms [42]. Research [43] has shown that compared to typically developing controls, ADHD patients exhibit significantly reduced FA in the genu of the left internal capsule and the left inferior fronto-occipital fasciculus. Longitudinal studies have demonstrated that developmental impairment in the internal capsule and corona radiata is evident not only during childhood in patients with attention-deficit/hyperactivity disorder but also that these developmental abnormalities persist into adulthood [44]. Moreover, MRI data combined with genomic analyses have revealed significant associations between FA values in the posterior limb of the left internal capsule and the left anterior corona radiata and the rs3908461 variant, a genetic risk factor for ADHD, in children aged 6–16 years [45].

### 3.4. Developmental Impairment in Other White Matter Tracts

The cingulum bundle, a core white matter pathway of the limbic system, connects the anterior cingulate cortex, posterior cingulate cortex, and hippocampus, and its developmental abnormalities are closely related to ADHD. Studies have shown that microstructural alterations in different segments of the cingulum bundle in children with ADHD are significantly associated with distinct symptom dimensions [37,46], and damage to the right cingulum angular bundle is particularly related to hyperactive–impulsive symptoms [23]. In addition, studies have found that, compared with typically developing children, children with ADHD exhibit significantly lower FA in the splenium of the bilateral corpus callosum, the left inferior fronto-occipital fasciculus, the bilateral inferior longitudinal fasciculus, and the bilateral parieto-occipital pontine tract [22].

Taken together, previous studies indicate that children with ADHD exhibit developmental impairment of white matter, with the main affected regions including the superior and inferior longitudinal fasciculi, corpus callosum, cingulum, and internal capsule. Furthermore, these white matter abnormalities are not confined to childhood; varying degrees of structural damage or atypicality are also present in adults with ADHD [21].

Although large-scale diffusion MRI studies identify disruptions within major white matter tracts such as the uncinate fasciculus and corpus callosum, some degree of heterogeneity remains across the literature. These discrepancies can be partly attributed to methodological variations in tractography and diffusion modeling techniques. A meta-analysis of 129 diffusion imaging studies found persistent FA reductions in the splenium and body of the corpus callosum in ADHD individuals. However, FA reductions correlated significantly with age, and group differences disappeared in pediatric-only analyses. Notably, 68% of studies were rated low-quality due to non-isotropic voxel acquisition or inadequate motion correction [19]. Using TBSS methodology, another meta-analysis showed age-related FA decline in the corpus callosum splenium across the ADHD lifespan [47]. A comparative meta-analysis of 28 ADHD and 23 ASD datasets revealed overlapping corpus callosum microstructural abnormalities, with shared deficits persisting from childhood to adulthood and showing progressive worsening [40].

Collectively, these findings underscore that clinicodemographic heterogeneity and methodological variations represent major barriers to consistency and comparability across studies investigating white matter alterations in ADHD. Standardization of acquisition protocols, analytical approaches, and participant characterization should be prioritized in future investigations to enhance the reliability and generalizability of research findings [19].

## 4. Features of the Functional Brain Network in Children with ADHD

Functional magnetic resonance imaging (fMRI) is a non-invasive technique that investigates brain activity by measuring blood-oxygen-level-dependent (BOLD) signals; it is categorized into resting-state fMRI (rs-fMRI) and task-based fMRI (task-fMRI). Existing studies suggest that, in the resting state, children with ADHD exhibit aberrant resting-state functional connectivity within the default mode network (DMN) and abnormal functional coupling between the striatum and frontal regions [25]. During task performance, specifically in response inhibition and cognitive processing tasks, children with ADHD show altered activation levels in regions such as the middle frontal gyrus, parahippocampal gyrus, and insula [48], as well as aberrant connectivity between the frontoparietal network and the ventral attention network [49].

### 4.1. Resting-State Functional Neuroimaging Studies

**Figure 1:**
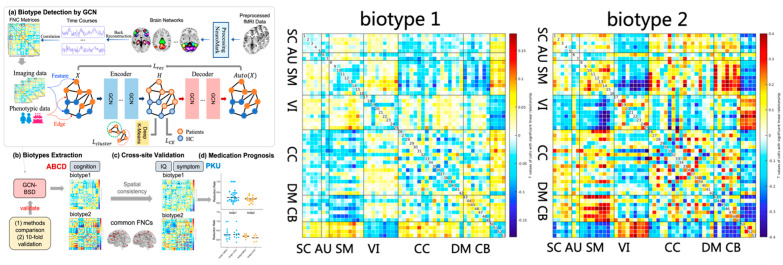
Two ADHD biotypes identified based on rs-fMRI features [51].

Large-scale sample studies reveal that children with ADHD show a distinct, often immature hierarchy of brain resting-state functional connectivity. This hierarchy is characterized by altered integration and segregation within and between key brain networks, reflecting both global and regional disruptions. Aberrant resting-state functional connectivity of the default mode network (DMN) is one of the abnormal features of resting-state neuroimaging in children with ADHD [50]. Large-scale studies have demonstrated that, at the whole-brain level, patients with ADHD exhibit reduced resting-state global connectivity in core hubs of the DMN, such as the posterior cingulate cortex/precuneus. At the network level, resting-state functional connectivity is weakened between the salience/attention network (SAN) and the sensorimotor and auditory networks in patients with ADHD [24]. Another study [49] reported hyperconnectivity among the DMN, attention networks, and the central executive network, whereas hypoconnectivity was observed between the visual attention network/sensorimotor network and the central executive network. At the regional level, large-scale studies demonstrate that, compared with typically developing controls, patients with ADHD show enhanced resting-state functional connectivity between the striatum and the temporal lobe, fronto-insular cortex, and supplementary motor area, as well as strengthened connectivity between the amygdala and the dorsal anterior cingulate cortex [25]. A large-scale study utilizing deep learning algorithms identified two distinct neurobiological biotypes in children with ADHD, as detailed in Figure 1. Biotype 1 was characterized by aberrant resting-state functional connectivity within the default mode network, as well as between the sensorimotor network and the cerebellum, and between the visual and subcortical networks. Biotype 2 exhibited disrupted resting-state functional connectivity between the DMN and the sensorimotor network, and between the visual network and the cerebellum. These biotypes represent emerging research constructs that require further clinical validation. However, the identification of these two neurobiological biotypes of ADHD holds promising potential for guiding precision pharmacotherapy by integrating patients’ neuroimaging features into treatment decision-making [51].

Overall, DMN dysfunction constitutes a salient neuroimaging characteristic of children with ADHD. Aberrant DMN functional connectivity is associated with transient attentional lapses, thereby compromising goal-directed behavior [52]. Ineffective suppression of DMN activity impairs selective attention [53], perhaps contributing to the core symptoms of inattention and hyperactivity in affected individuals.

### 4.2. Task-Based Functional Neuroimaging Studies

Deficits in inhibitory control may constitute one of the salient functional neuroimaging features in patients with ADHD [54]. During the stop-signal task, children with ADHD exhibit significantly reduced functional connectivity within the response inhibition network compared to controls, while functional connectivity among DMN regions is significantly elevated [55]. Data from the Adolescent Brain Cognitive Development study indicate that children with ADHD and comorbid irritability demonstrate lower activation in frontoparietal regions associated with response inhibition during the stop-signal task relative to typically developing controls [27]. In the Go/No-Go task, children with ADHD show reduced activation extent within the DMN, dorsal attention network, and limbic network [26]. Additionally, during cognitive processing tasks, research has found that activation in the middle frontal gyrus, parahippocampal gyrus, and insula is significantly lower in children with ADHD compared to controls [48], as detailed in Table 1.

## 5. Discussion

This review possesses several strengths that enhance its academic and clinical value. It focuses exclusively on large-scale MRI studies, which effectively mitigates the low reproducibility commonly observed in small-sample neuroscience research, thereby ensuring that the synthesized findings are more reliable. This review adopts a multimodal perspective by integrating evidence from structural, diffusion, and functional MRI, providing a comprehensive understanding of the neurobiological underpinnings of ADHD.

Despite its strengths, some potential limitations should be acknowledged. First, this review does not explicitly address the heterogeneity among included large-scale studies, such as variations in ADHD diagnostic criteria, participant age ranges, comorbid conditions, and MRI scanning/analysis protocols [19]. These factors may influence the consistency of neuroimaging findings and limit the generalizability of the synthesized conclusions [56]. Second, this review focuses primarily on cross-sectional large-scale studies, with limited integration of longitudinal research evidence.

Building on the findings and limitations of this review, future research should prioritize several directions. First, to address the heterogeneous nature of neuroimaging features in children with ADHD, future studies should strengthen international collaboration through large-scale consortia and open-access datasets such as ADHD-200, ENIGMA-ADHD [12], and the Adolescent Brain Cognitive Development (ABCD) Study^®^. These efforts will enhance statistical power and improve the generalizability of research findings. Second, advancing the identification and characterization of ADHD neurobiological subtypes is necessary [57,58]. Stratifying patients based on distinct neural phenotypes and integrating these insights with clinical pharmacotherapy holds promise for optimizing treatment efficacy and advancing the field toward precision medicine [51,59]. Third, large-scale longitudinal studies are needed to elucidate developmental trajectories of brain alterations in ADHD.

## 6. Conclusions

This narrative review synthesizes evidence from large-scale multimodal neuroimaging studies, highlighting distinct patterns of neurodevelopmental alterations in children with ADHD. The reviewed literature suggests that ADHD is associated with atypical cortical and subcortical development, widespread white matter microstructural disruptions, and altered resting-state functional connectivity within the default mode network. These neural features show promise as potential candidate biomarkers for improving diagnostic accuracy and guiding personalized interventions.

## Data Availability

No new data were created or analyzed in this study.

## Abbreviations

The following abbreviations are used in this manuscript:

ADHD	Attention-Deficit/Hyperactivity Disorder
ADHD-C	ADHD combined subtype
ADHD-I	ADHD predominantly inattentive subtype
MRI	Magnetic resonance imaging
sMRI	Structural magnetic resonance imaging
dMRI	Diffusion magnetic resonance imaging
fMRI	Functional magnetic resonance imaging
rs-fMRI	resting-state functional magnetic resonance imaging
FA	Fractional anisotropy
MD	Mean diffusivity
DMN	Default mode network
ABCD	Adolescent Brain Cognitive Development study

## References

[1] Posner J., Polanczyk G.V., Sonuga-Barke E. (2020). Attention-deficit hyperactivity disorder. Lancet.

[2] Koirala S., Grimsrud G., Mooney M.A., Larsen B., Feczko E., Elison J.T., Nelson S.M., Nigg J.T., Tervo-Clemmens B., Fair D.A. (2024). Neurobiology of attention-deficit hyperactivity disorder: Historical challenges and emerging frontiers. Nat. Rev. Neurosci..

[3] Salari N., Ghasemi H., Abdoli N., Rahmani A., Shiri M.H., Hashemian A.H., Akbari H., Mohammadi M. (2023). The global prevalence of ADHD in children and adolescents: A systematic review and meta-analysis. Ital. J. Pediatr..

[4] Song P., Zha M., Yang Q., Zhang Y., Li X., Rudan I. (2021). The prevalence of adult attention-deficit hyperactivity disorder: A global systematic review and meta-analysis. J. Glob. Health.

[5] Platania N.M., Starreveld D.E.J., Wynchank D., Beekman A.T.F., Kooij S. (2025). Bias by gender: Exploring gender-based differences in the endorsement of ADHD symptoms and impairment among adult patients. Front. Glob. Womens Health.

[6] Liu J., Liu Q.R., Wu Z.M., Chen Q.R., Chen J., Wang Y., Cao X.L., Dai M.X., Dong C., Liu Q. (2023). Specific brain imaging alterations underlying autistic traits in children with attention-deficit/hyperactivity disorder. Behav. Brain Funct..

[7] Liu Q., Feng Y., Chen W., Zhu Y., Preece D.A., Gao Y., Luo X., Dang C., Wang Y., Sun L. (2025). Emotion regulation strategy and its relationship with emotional dysregulation in children with attention-deficit/hyperactivity disorder: Behavioral and brain findings. Eur. Child. Adolesc. Psychiatry.

[8] Marek S., Tervo-Clemmens B., Calabro F.J., Montez D.F., Kay B.P., Hatoum A.S., Donohue M.R., Foran W., Miller R.L., Hendrickson T.J. (2022). Reproducible brain-wide association studies require thousands of individuals. Nature.

[9] Turner B.O., Paul E.J., Miller M.B., Barbey A.K. (2018). Small sample sizes reduce the replicability of task-based fMRI studies. Commun. Biol..

[10] Zuo X.N., Xu T., Milham M.P. (2019). Harnessing reliability for neuroscience research. Nat. Hum. Behav..

[11] Button K.S., Ioannidis J.P., Mokrysz C., Nosek B.A., Flint J., Robinson E.S., Munafò M.R. (2013). Power failure: Why small sample size undermines the reliability of neuroscience. Nat. Rev. Neurosci..

[12] Hoogman M., Muetzel R., Guimaraes J.P., Shumskaya E., Mennes M., Zwiers M.P., Jahanshad N., Sudre G., Wolfers T., Earl E.A. (2019). Brain Imaging of the Cortex in ADHD: A Coordinated Analysis of Large-Scale Clinical and Population-Based Samples. Am. J. Psychiatry.

[13] Shaw P., Eckstrand K., Sharp W., Blumenthal J., Lerch J.P., Greenstein D., Clasen L., Evans A., Giedd J., Rapoport J.L. (2007). Attention-deficit/hyperactivity disorder is characterized by a delay in cortical maturation. Proc. Natl. Acad. Sci. USA.

[14] Hoogman M., Bralten J., Hibar D.P., Mennes M., Zwiers M.P., Schweren L.S.J., van Hulzen K.J.E., Medland S.E., Shumskaya E., Jahanshad N. (2017). Subcortical brain volume differences in participants with attention deficit hyperactivity disorder in children and adults: A cross-sectional mega-analysis. Lancet Psychiatry.

[15] Giedd J.N. (2019). The Enigma of Neuroimaging in ADHD. Am. J. Psychiatry.

[16] Bernanke J., Luna A., Chang L., Bruno E., Dworkin J., Posner J. (2022). Structural brain measures among children with and without ADHD in the Adolescent Brain and Cognitive Development Study cohort: A cross-sectional US population-based study. Lancet Psychiatry.

[17] Mu S., Wu H., Zhang J., Chang C. (2022). Structural Brain Changes and Associated Symptoms of ADHD Subtypes in Children. Cereb. Cortex.

[18] Usha Rupni K., Aruna Priya P. (2023). Identification of Attention-Deficit-Hyperactivity Disorder Subtypes Based on Structural MRI Grey Matter Volume and Phenotypic Information. Curr. Med. Imaging.

[19] Parlatini V., Itahashi T., Lee Y., Liu S., Nguyen T.T., Aoki Y.Y., Forkel S.J., Catani M., Rubia K., Zhou J.H. (2023). White matter alterations in Attention-Deficit/Hyperactivity Disorder (ADHD): A systematic review of 129 diffusion imaging studies with meta-analysis. Mol. Psychiatry.

[20] Sudre G., Norman L., Bouyssi-Kobar M., Price J., Shastri G.G., Shaw P. (2023). A Mega-analytic Study of White Matter Microstructural Differences Across 5 Cohorts of Youths With Attention-Deficit/Hyperactivity Disorder. Biol. Psychiatry.

[21] Fuelscher I., Hyde C., Thomson P., Vijayakumar N., Sciberras E., Efron D., Anderson V., Hazell P., Silk T.J. (2023). Longitudinal Trajectories of White Matter Development in Attention-Deficit/Hyperactivity Disorder. Biol. Psychiatry Cogn. Neurosci. Neuroimaging.

[22] Fuelscher I., Hyde C., Anderson V., Silk T.J. (2021). White matter tract signatures of fiber density and morphology in ADHD. Cortex.

[23] Damatac C.G., Chauvin R.J.M., Zwiers M.P., van Rooij D., Akkermans S.E.A., Naaijen J., Hoekstra P.J., Hartman C.A., Oosterlaan J., Franke B. (2022). White Matter Microstructure in Attention-Deficit/Hyperactivity Disorder: A Systematic Tractography Study in 654 Individuals. Biol. Psychiatry Cogn. Neurosci. Neuroimaging.

[24] Gao Y., Zhou Z., Bao W., Chen Y., Hu X., Li H., Zhang L., Gong Q., Huang X. (2025). Multiscale functional connectivity reveals imbalanced interplay between higher- and lower-order brain networks in ADHD. Nat. Ment. Health.

[25] Norman L.J., Sudre G., Price J., Shaw P. (2024). Subcortico-Cortical Dysconnectivity in ADHD: A Voxel-Wise Mega-Analysis Across Multiple Cohorts. Am. J. Psychiatry.

[26] Kim S.J., Tanglay O., Chong E.H.N., Young I.M., Fonseka R.D., Taylor H., Nicholas P., Doyen S., Sughrue M.E. (2023). Functional connectivity in ADHD children doing Go/No-Go tasks: An fMRI systematic review and meta-analysis. Transl. Neurosci..

[27] Lee K.S., Xiao J., Luo J., Leibenluft E., Liew Z., Tseng W.L. (2022). Characterizing the Neural Correlates of Response Inhibition and Error Processing in Children With Symptoms of Irritability and/or Attention-Deficit/Hyperactivity Disorder in the ABCD Study^®^. Front. Psychiatry.

[28] Albajara Sáenz A., Villemonteix T., Massat I. (2019). Structural and functional neuroimaging in attention-deficit/hyperactivity disorder. Dev. Med. Child Neurol..

[29] Rosch K.S., Thapaliya G., Plotkin M., Mostofsky S.H., Carnell S. (2024). Shared and distinct alterations in brain morphology in children with ADHD and obesity: Reduced cortical surface area in ADHD and thickness in overweight/obesity. J. Psychiatr. Res..

[30] Wang Y., Ma L., Wang J., Ding Y., Liu N., Men W., Tan S., Gao J.H., Qin S., He Y. (2024). The neural and genetic underpinnings of different developmental trajectories of Attention-Deficit/Hyperactivity Symptoms in children and adolescents. BMC Med..

[31] Ágrez K., Vakli P., Weiss B., Vidnyánszky Z., Bunford N. (2025). Assessing the association between ADHD and brain maturation in late childhood and emotion regulation in early adolescence. Transl. Psychiatry.

[32] Caye A., Rocha T.B., Anselmi L., Murray J., Menezes A.M., Barros F.C., Gonçalves H., Wehrmeister F., Jensen C.M., Steinhausen H.C. (2016). Attention-Deficit/Hyperactivity Disorder Trajectories From Childhood to Young Adulthood: Evidence From a Birth Cohort Supporting a Late-Onset Syndrome. JAMA Psychiatry.

[33] Moffitt T.E., Houts R., Asherson P., Belsky D.W., Corcoran D.L., Hammerle M., Harrington H., Hogan S., Meier M.H., Polanczyk G.V. (2015). Is Adult ADHD a Childhood-Onset Neurodevelopmental Disorder? Evidence From a Four-Decade Longitudinal Cohort Study. Am. J. Psychiatry.

[34] Agnew-Blais J.C., Polanczyk G.V., Danese A., Wertz J., Moffitt T.E., Arseneault L. (2016). Evaluation of the Persistence, Remission, and Emergence of Attention-Deficit/Hyperactivity Disorder in Young Adulthood. JAMA Psychiatry.

[35] Shamir I., Assaf Y. (2025). Tutorial: A guide to diffusion MRI and structural connectomics. Nat. Protoc..

[36] Lope-Piedrafita S. (2018). Diffusion Tensor Imaging (DTI). Methods Mol. Biol..

[37] Cooper M., Thapar A., Jones D.K. (2015). ADHD severity is associated with white matter microstructure in the subgenual cingulum. NeuroImage Clin..

[38] Aoki Y., Cortese S., Castellanos F.X. (2018). Research Review: Diffusion tensor imaging studies of attention-deficit/hyperactivity disorder: Meta-analyses and reflections on head motion. J. Child Psychol. Psychiatry.

[39] Chiang H.L., Wu C.S., Chen C.L., Tseng W.I., Gau S.S. (2024). Machine-learning-based feature selection to identify attention-deficit hyperactivity disorder using whole-brain white matter microstructure: A longitudinal study. Asian J. Psychiatr..

[40] Zhang K., Fu Z., Lai Q., Zhao Y., Liu J., Cao Q. (2023). The shared white matter developmental trajectory anomalies of attention-deficit/hyperactivity disorder and autism spectrum disorders: A meta-analysis of diffusion tensor imaging studies. Prog. Neuro-Psychopharmacol. Biol. Psychiatry.

[41] Rubia K. (2018). Cognitive Neuroscience of Attention Deficit Hyperactivity Disorder (ADHD) and Its Clinical Translation. Front. Hum. Neurosci..

[42] Connaughton M., Whelan R., O’Hanlon E., McGrath J. (2022). White matter microstructure in children and adolescents with ADHD. NeuroImage Clin..

[43] Zhou R., Dong P., Chen S., Qian A., Tao J., Zheng X., Cheng J., Yang C., Huang X., Wang M. (2022). The long-range white matter microstructural alterations in drug-naive children with ADHD: A tract-based spatial statistics study. Psychiatry Res. Neuroimaging.

[44] Cortese S., Imperati D., Zhou J., Proal E., Klein R.G., Mannuzza S., Ramos-Olazagasti M.A., Milham M.P., Kelly C., Castellanos F.X. (2013). White matter alterations at 33-year follow-up in adults with childhood attention-deficit/hyperactivity disorder. Biol. Psychiatry.

[45] Fu G.H., Chen W., Li H.M., Wang Y.F., Liu L., Qian Q.J. (2021). A potential association of RNF219-AS1 with ADHD: Evidence from categorical analysis of clinical phenotypes and from quantitative exploration of executive function and white matter microstructure endophenotypes. CNS Neurosci. Ther..

[46] Chiang H.L., Tseng W.I., Tseng W.L., Tung Y.H., Hsu Y.C., Chen C.L., Gau S.S. (2023). Atypical development in white matter microstructures in ADHD: A longitudinal diffusion imaging study. Asian J. Psychiatr..

[47] Cui J., Wang Z., Xu Y., Li Y., Yu H., Ping L., Jin S., Cheng Y., Xu X., Zhang Y. (2023). Altered White Matter Integrity in ADHD Revealed by Meta-analysis of Tract-based Spatial Statistics. J. Atten. Disord..

[48] Tamon H., Fujino J., Itahashi T., Frahm L., Parlatini V., Aoki Y.Y., Castellanos F.X., Eickhoff S.B., Cortese S. (2024). Shared and Specific Neural Correlates of Attention Deficit Hyperactivity Disorder and Autism Spectrum Disorder: A Meta-Analysis of 243 Task-Based Functional MRI Studies. Am. J. Psychiatry.

[49] Gao Y., Shuai D., Bu X., Hu X., Tang S., Zhang L., Li H., Hu X., Lu L., Gong Q. (2019). Impairments of large-scale functional networks in attention-deficit/hyperactivity disorder: A meta-analysis of resting-state functional connectivity. Psychol. Med..

[50] Norman L.J., Sudre G., Price J., Shastri G.G., Shaw P. (2023). Evidence from “big data” for the default-mode hypothesis of ADHD: A mega-analysis of multiple large samples. Neuropsychopharmacology.

[51] Feng A., Zhi D., Feng Y., Jiang R., Fu Z., Xu M., Zhao M., Yu S., Stevens M., Sun L. (2024). Functional imaging derived ADHD biotypes based on deep clustering: A study on personalized medication therapy guidance. EClinicalMedicine.

[52] Menon V. (2023). 20 years of the default mode network: A review and synthesis. Neuron.

[53] Weissman D.H., Roberts K.C., Visscher K.M., Woldorff M.G. (2006). The neural bases of momentary lapses in attention. Nat. Neurosci..

[54] Senkowski D., Ziegler T., Singh M., Heinz A., He J., Silk T., Lorenz R.C. (2024). Assessing Inhibitory Control Deficits in Adult ADHD: A Systematic Review and Meta-analysis of the Stop-signal Task. Neuropsychol. Rev..

[55] Kowalczyk O.S., Mehta M.A., O’Daly O.G., Criaud M. (2022). Task-Based Functional Connectivity in Attention-Deficit/Hyperactivity Disorder: A Systematic Review. Biol. Psychiatry Glob. Open Sci..

[56] van Ewijk H., Heslenfeld D.J., Zwiers M.P., Buitelaar J.K., Oosterlaan J. (2012). Diffusion tensor imaging in attention deficit/hyperactivity disorder: A systematic review and meta-analysis. Neurosci. Biobehav. Rev..

[57] Wang Q., Zhao C., Qiu J., Lu W. (2023). Two neurosubtypes of ADHD different from the clinical phenotypes. Psychiatry Res..

[58] Luo N., Luo X., Zheng S., Yao D., Zhao M., Cui Y., Zhu Y., Calhoun V.D., Sun L., Sui J. (2023). Aberrant brain dynamics and spectral power in children with ADHD and its subtypes. Eur. Child Adolesc. Psychiatry.

[59] Hu L.F., Zhong Y.Y., Wang P., Liu L., Cao X.L., Sun L., Cao Q.J., Yang L., Qian Y., Wang Y.F. (2025). White matter microstructural subgroups of children with ADHD: Similar clinical presentations and distinct neuropsychological profiles. J. Psychiatr. Res..

